# Comparison Between Phenotypic Profile and Functional Aspects of IL‐9‐Producing Lymphocytes, Th17 and Tfh of Individuals From Endemic and Non‐Endemic Areas for Hookworm Infection

**DOI:** 10.1111/pim.70070

**Published:** 2026-03-13

**Authors:** Yvanna Louise Di Christine Oliveira, Marcelo Eduardo Cardozo, Luisa Mourão Dias Magalhães, Carlos Thailan de Jesus Santos, Ramayana Morais de Medeiros Brito, Luciana Maria de Oliveira, Ana Carolina Amado Gomes, Vinícius Torres Castro Campos, Ricardo Toshio Fujiwara, Silvio Santana Dolabella, Lilian Lacerda Bueno

**Affiliations:** ^1^ Laboratory of Tropical Entomology and Parasitology, Postgraduate Program in Pharmaceutical Sciences Federal University of Sergipe São Cristóvão Brazil; ^2^ Laboratory of Immunobiology and Parasite Control, Department of Parasitology Federal University of Minas Gerais Belo Horizonte Brazil; ^3^ Laboratory of Interactions in ImmunoParasitology, Department of Parasitology Federal University of Minas Gerais Belo Horizonte Brazil; ^4^ Laboratory of Tropical Entomology and Parasitology, Postgraduate Program in Parasite Biology Federal University of Sergipe São Cristóvão Brazil

**Keywords:** follicular T‐helper cells, helminthiasis, hookworm infection, IL‐9, T‐helper lymphocytes

## Abstract

Hookworm infections remain a major public health concern in endemic areas, modulating both the adaptive and innate immune systems. While the type 2 response is well‐characterised, the roles of T follicular helper (Tfh), Th17, and IL‐9‐producing lymphocytes remain poorly defined. Here, we characterised these T cell subsets in individuals naturally infected with hookworms. From 1500 faecal samples screened, 60 were positive for hookworms, and peripheral blood was collected from 10 uninfected controls from endemic (NEG END) and non‐endemic (NEG NE) areas, as well as from 7 infected individuals before (HKW BT) and after (HKW PT) treatment. Infected individuals displayed haematological alterations, including anaemia (*n* = 2), eosinophilia (*n* = 1), monocytosis (*n* = 4), and lymphocytosis (*n* = 3), along with an expansion of PBMCs, particularly Tfh cells, during infection. Expression of IL‐9 and IL‐10 by Tfh cells was markedly elevated after treatment. In contrast, individuals from non‐endemic areas displayed a distinct baseline profile with higher Tfh activation (CD69) expression, suggesting immune adaptation in endemic settings. While IL‐10‐producing Tfh expanded during infection, IL‐9‐producing cells and Th17 cells expanded mainly after treatment. These findings suggest that individuals living in endemic areas, regardless of infection status, exhibit signs of persistent antigenic stimulation that promote a more tolerogenic and regulated immune profile. Moreover, hookworm infection and subsequent treatment reshape the immune landscape, highlighting the contribution of Tfh‐ and Th17‐associated pathways, as well as IL‐9 and IL‐10 production, in modulating host–parasite interactions.

## 1 Introduction

Hookworms, together with *Trichuris trichiura*, *Strongyloides stercoralis*, and *Ascaris lumbricoides*, comprise a group of human parasitic infections known as soil‐transmitted helminths. Hookworms are parasitic nematodes that, during their life cycle, exhibit larvae that migrate through various host tissues. This migration elicits a systemic immune response, predominantly type 2, which is crucial for maintaining homeostatic balance and mitigating host damage by promoting tolerance to the parasite [1, 2].

The type 2 immune response is characterised by an increase in the number of effector cells producing interleukins (IL‐4, IL‐5, and IL‐13), such as Th2 cells, type 2 innate lymphoid cells (ILC2s), natural killer (NK) cells, eosinophils, basophils, and mast cells [3, 4, 5]. These cells collectively aim to enhance mucus production by intestinal epithelial cells and augment the host's peristaltic functions, thereby facilitating worm expulsion and aiding in the reduction of immunopathogenesis, which subsequently decreases host tissue damage [6, 7].

Lymphocytes possess the ability to adapt to environmental changes and modulate their responses to varying conditions and pathogens through epigenetic alterations [8, 9]. Although the type 2 immune response is well‐characterised and has been extensively elucidated in recent years, it is understood that Th2 lymphocytes are not the sole contributors to this response. Th9 lymphocytes have been studied for their predominantly inflammatory role, which is essential for regulating parasite numbers [10].

Th cells differentiate into Th9 cells upon stimulation with transforming growth factor (TGF‐β) and IL‐4 and are characterised as CD4^+^IL‐4^−^IL‐9^+^IL‐10^+^. These cells, in concert with Th2 cells, promote a type 2 inflammatory response that, unlike the type 1 response, induces tolerance and resistance to the parasite while preventing excessive proliferation [10, 11]. In turn, T follicular helper cells (Tfh) characterised by the expression of the transcription factors B‐cell lymphoma 6 protein (Bcl6), CXC chemokine receptor type 5 (CXCR5), and inducible T‐cell costimulator (ICOS), as well as the secretion of interleukin 21 (IL‐21), are responsible for the differentiation of B cells and their migration to the germinal centre, where they initiate antibody production [12, 13, 14].

Th17 cells are classically recognised for their involvement in the defence against fungal and bacterial infections and are also implicated in immune responses associated with autoimmune and allergic diseases [15]. These cells are characterised by the production of IL‐17A, IL‐17F, and IL‐22, and their differentiation is driven by the transcription factors RORγt, CCR6, and STAT3. Although commonly associated with Th1 cells, Th17 cells exhibit plasticity [16, 17] and can adopt a Th2‐like phenotype in helminth infections and asthma [15, 18].

Given the limited information available on the immune response in hookworm‐infected patients, the objective of this study was to evaluate the presence and involvement of IL‐9‐producing cells, Th17, and Tfh cells in hookworm‐infected or uninfected individuals from both endemic and non‐endemic areas before or after treatment.

## 2 Methods

### 2.1 Study Design, Population and Ethical Statement

**FIGURE 1:**
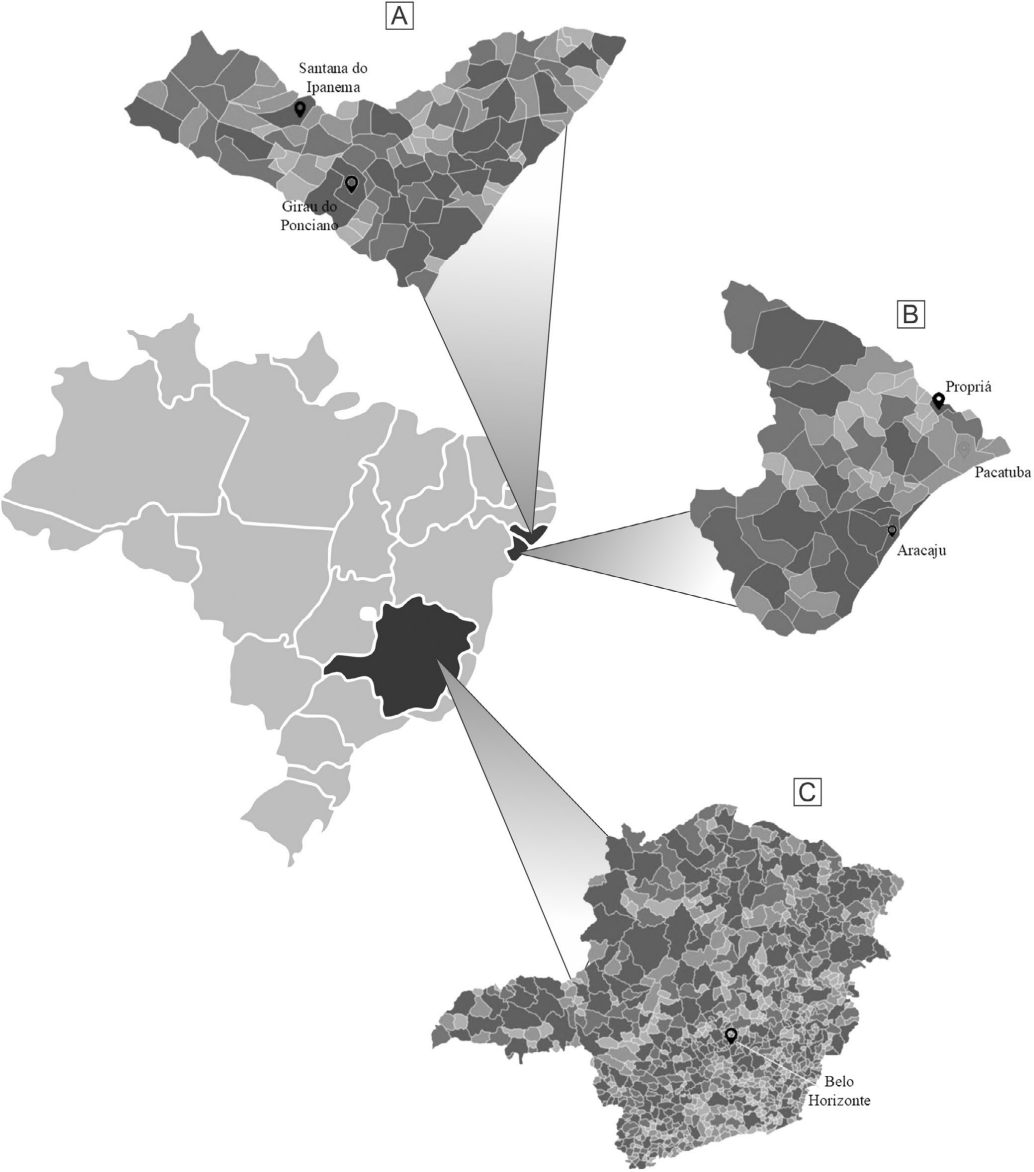
Maps of the Alagoas (A), Sergipe (B), and Minas Gerais states (C), Brazil, with the collection municipalities marked with a red locator.

This prospective cross‐sectional study was conducted in two distinct geographical regions in Brazil (Figure 1). The endemic area encompasses rural and semi‐rural municipalities in the states of Sergipe (Propriá, Aracaju, and Pacatuba) and Alagoas (Santana do Ipanema and Girau do Ponciano), Northeastern Brazil, characterised by lower urbanisation indices and historical transmission of soil‐transmitted helminths. The non‐endemic area is Belo Horizonte (Minas Gerais), a highly urbanised state capital in the Southeast region which has been non‐endemic for hookworm or other soil‐transmitted helminths since the 20th century [19, 20]. Participants were selected for faecal and peripheral blood sample collection from July 2020 to March 2023. Demographic and socioeconomic characteristics of the included population are summarised in Supplementary Table S1. The non‐endemic negative group consisted of healthy volunteers (participants in this group were interviewed and reported no history of past helminthic infections) from Belo Horizonte with a mean age of 28.3 years (53.8% female). The endemic negative group comprised individuals from the endemic region who tested negative for helminths, with a mean age of 25.8 years (40% female). The hookworm infected group included individuals with confirmed mono‐infection, with a mean age of 25.1 years (40% female). The Ethics Committee of the Federal University of Sergipe approved this study (CAAE 66074017.6.1001.5546).

### 2.2 Determination of Infection and Treatment of Infected Individuals

To identify hookworm infections, faecal parasitological examinations were conducted using Ritchie's method (formalin‐ether concentration) [21]. This method was selected over Kato‐Katz due to logistical considerations; the use of formalin allowed for the preservation of stool samples during transport from collection sites to the laboratory, preventing egg degradation that could occur with delayed Kato‐Katz readings. Parasite load was not quantified. All infected patients (HKW BT) were treated with albendazole, and 30 days post‐treatment, new blood and faecal samples were collected for post‐treatment parasitological and immunological analyses (HKW PT). Additionally, individuals with negative egg counts from endemic (NEG END) and non‐endemic (NEG NE) areas provided blood samples and were included in this study as negative controls. Strict inclusion criteria were applied to ensure a mono‐infected cohort. Samples were screened for the presence of other common soil‐transmitted helminths and *S mansoni*. Individuals presenting with any co‐infection were excluded from the study.

### 2.3 Obtaining Peripheral Blood Mononuclear Cells (PBMC)

PBMCs were isolated from heparinized blood by density gradient centrifugation (Histopaque, Sigma Aldrich Co., USA). Cells were washed twice with RPMI 1640 medium (Invitrogen Co., USA) supplemented with 10% foetal bovine serum (FBS) at 4°C for 10 min. Following isolation, PBMCs were resuspended in FBS containing 10% DMSO and cryopreserved in liquid nitrogen until use. To ensure sample integrity and minimise batch effects, all cryopreserved samples from the endemic area were consolidated and transported to the central laboratory in Belo Horizonte in a single shipment using liquid nitrogen dry shippers. For analysis, cells were thawed, washed, and resuspended at a final concentration of 1 × 10^7^ cells/mL in RPMI 1640 medium [22].

### 2.4 Identification of T Helper Cells Subpopulations

**FIGURE 2:**
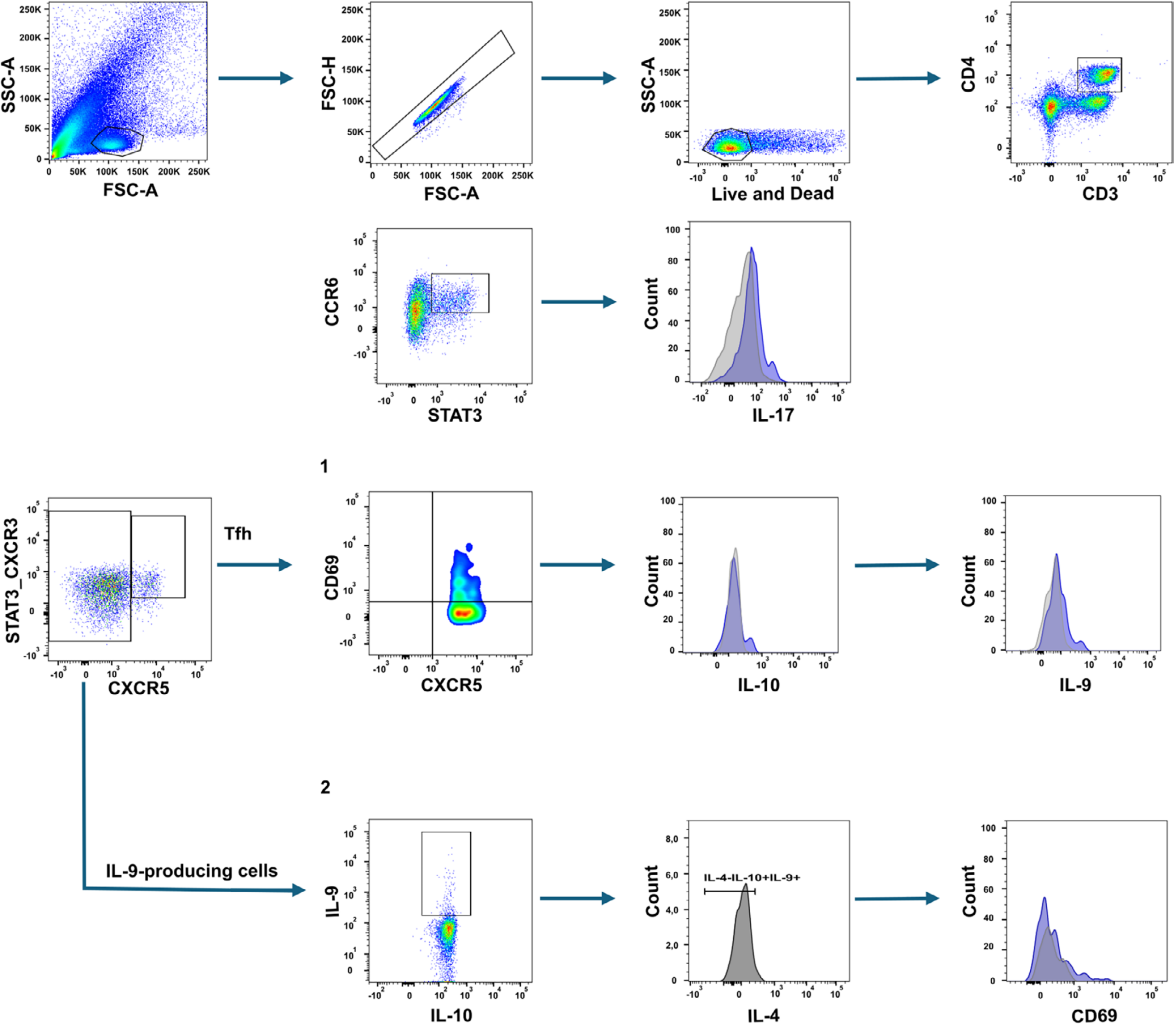
Gating strategy for identification of T cells. (A) Manual gating strategy for the identification of CD3^+^CD4^+^ cells, (B) Th17 cells, (C1) Tfh cells, and (C2) IL‐9‐producing cells. Grey markings show negative control. Purple markings show positive samples.

To identify subpopulations of T‐helper cells, cryopreserved PBMCs were thawed. As a positive control to validate intracellular cytokine detection capacity, a subset of cells was stimulated with PMA (Phorbol 12‐myristate 13‐acetate; 50 ng/mL) and Ionomycin (1 μg/mL) in the presence of a protein transport inhibitor (Brefeldin A; 10 μg/mL) for 4 h. For the experimental phenotypic analysis, cells were incubated with a viability dye to exclude dead cells. After washing, 20 μL of monoclonal antibodies targeting surface markers were added. The cells were then fixed with 4% formaldehyde and permeabilized with 0.05% saponin, followed by the addition of monoclonal antibodies for intracellular markers (Table 1). Isotype controls were included to evaluate non‐specific staining, and negative controls were used to set gating boundaries. To ensure accurate compensation, compensation beads (BD CompBeads; Becton Dickinson) were utilised for all fluorochromes. Data acquisition was performed on a single LSR Fortessa cytometer (Becton Dickinson, USA) at the central laboratory to eliminate instrument variability, acquiring at least 150,000 events per sample. Data were analysed using FlowJo software (Tree Star Inc., USA) (Figure 2).

**TABLE 1:** Surface and intracellular markers with their respective fluorochromes for identification of Tfh, Th17 and Th9 cells.

Location	Marker	Fluorochrome	Clone	Cell
	Viability reagent	Alexa700	—	Th9/Th17/Tfh
Surface	CD4	FITC	RPA‐T4	Th9/Tfh
	CXCR5	PERCP‐Cy5.5	RF8B2	Tfh
	CD69	PE‐Cy7	FN50	Th9/Tfh
	CCR4	BV605	BV605	Th17
	CCR6	PERCP	11A9	Th17
Intracell	CD3	APC‐Cy7	SK7	Th9/Tfh
	IL‐4	PE	8D4‐8	Th9
	IL‐9	BV421	MH9A3	Th9
	IL‐10	PECF	JES3‐19F1	Th9/Tfh
	IL‐17	BV421	N49‐653	Th17
	STAT3	AF647	Py705	Tfh/Th17

### 2.5 Statistical Analysis

Multivariate data analysis was conducted to determine the association between immunological aspects triggered by infection and parasitological factors defining the morbidity of hookworm infection. The obtained data were analysed using the GraphPad Prism statistical package, version 8.0.2. The Shapiro–Wilk test for normality was applied. Potential outliers were identified and excluded using the Grubbs' test, followed by the Mann–Whitney test (for non‐normally distributed and unpaired data) or the Wilcoxon test (for non‐normally distributed and paired data).

## 3 Results

From 2019 to 2023, faecal samples were collected from 1500 patients in endemic areas for hookworms in the Brazilian states of Sergipe and Alagoas, as well as from a non‐endemic area in the state of Minas Gerais. Overall, 4% (60/1500) of the participants tested positive for hookworm infection. Among these, 43 individuals consented to provide peripheral blood samples and were included in the pre‐treatment hookworm‐infected group (HKW BT). Of this initial cohort, 12 individuals completed anthelmintic treatment and returned for blood collection 30 days post‐treatment, constituting the post‐treatment group (HKW PT).

**FIGURE 3:**
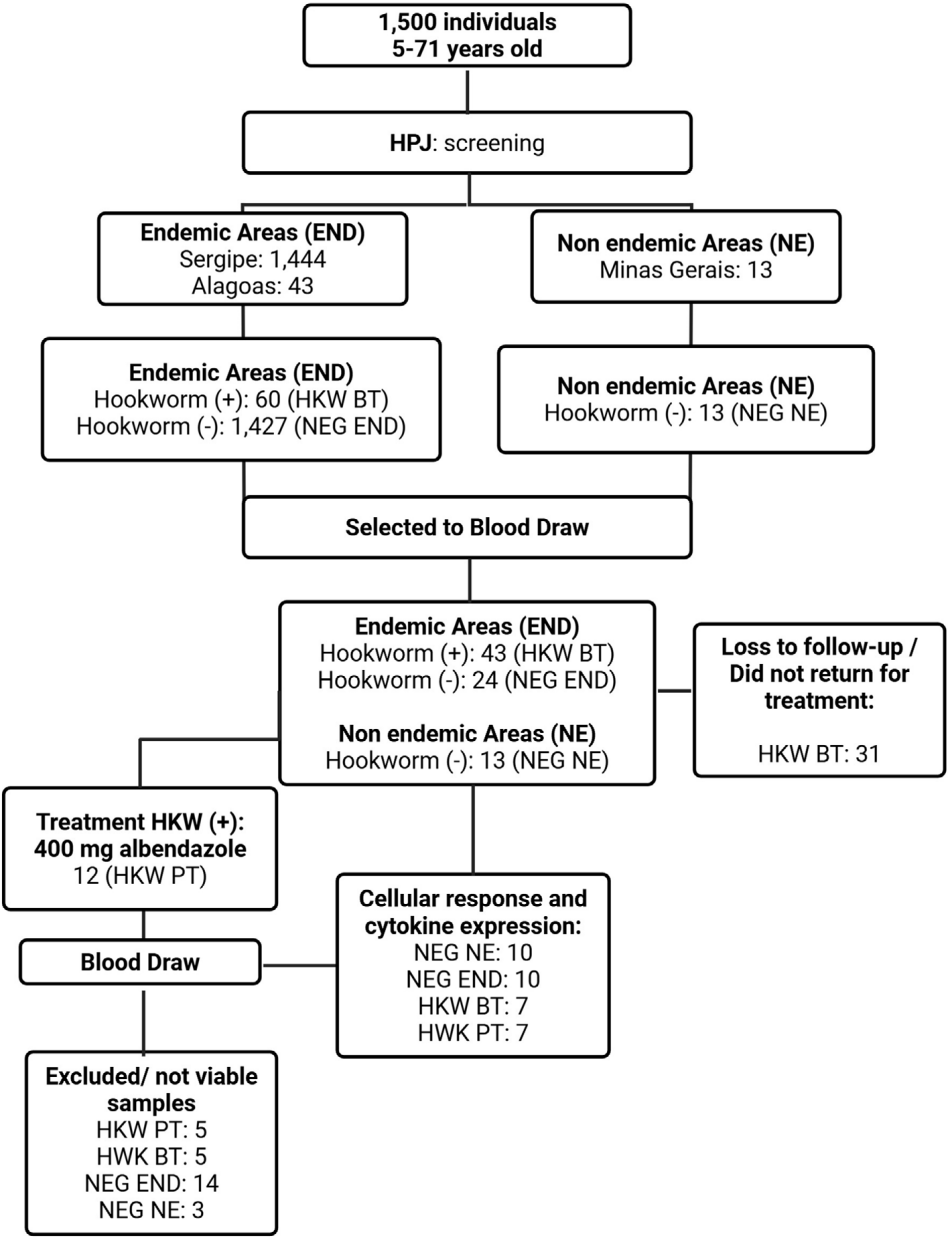
Study design. A total of 1500 stool samples were analysed from individuals living in hookworm‐endemic and non‐endemic areas, of which 60 tested positives for hookworm infection. Among infected individuals, 43 provided blood samples prior to treatment (HKW BT), and 12 completed anthelmintic therapy and returned for blood collection 30 days post‐treatment (HKW PT). In parallel, egg‐negative individuals from endemic (NEG END) and non‐endemic (NEG NE) areas were recruited as controls. Following application of a strict post‐thaw PBMC viability cutoff (≥ 70%), the final number of samples included in flow cytometry analyses was NEG NE (*n* = 10), NEG END (*n* = 10), HKW BT (*n* = 7), and HKW PT (*n* = 7).

In parallel, blood samples were obtained from 24 egg‐negative individuals living in endemic areas (NEG END) and from 13 egg‐negative individuals from the non‐endemic area (NEG NE), who served as negative control groups (Figure 3).

For all flow cytometry analyses, a stringent post‐thaw viability cutoff of 70% was applied to peripheral blood mononuclear cell (PBMC) samples. Samples failing to meet this criterion were excluded from downstream analyses to ensure data quality and reliability. After exclusion of non‐viable samples, the final number of individuals included in the study was as follows: NEG NE (*n* = 10), NEG END (*n* = 10), HKW BT (*n* = 7), and HKW PT (*n* = 7).

### 3.1 Pati ents With Hookworm Infection Exhibited Monocytosis and Lymphocytosis, but Not Eosinophilia

To evaluate the systemic haematological impact of hookworm infection, we performed a complete blood count analysis on all participants. Peripheral blood samples were collected in EDTA tubes for complete blood count (CBC) analysis. Among the hookworm‐infected patients, only two presented mildly reduced haemoglobin levels (12.3 and 10.3 g/dL), and just one displayed a slight increase in eosinophil count (380/mm^3^). In contrast, four patients showed monocytosis and three exhibited lymphocytosis. Following anthelmintic treatment, no haematological abnormalities were detected at follow‐up, and previously observed anaemia, monocytosis, and lymphocytosis were resolved (Supporting Information S1).

### 3.2 Hookworm Infection Promotes Expansion but Dampens Activation of T Follicular Helper (Tfh) Cells

**FIGURE 4:**
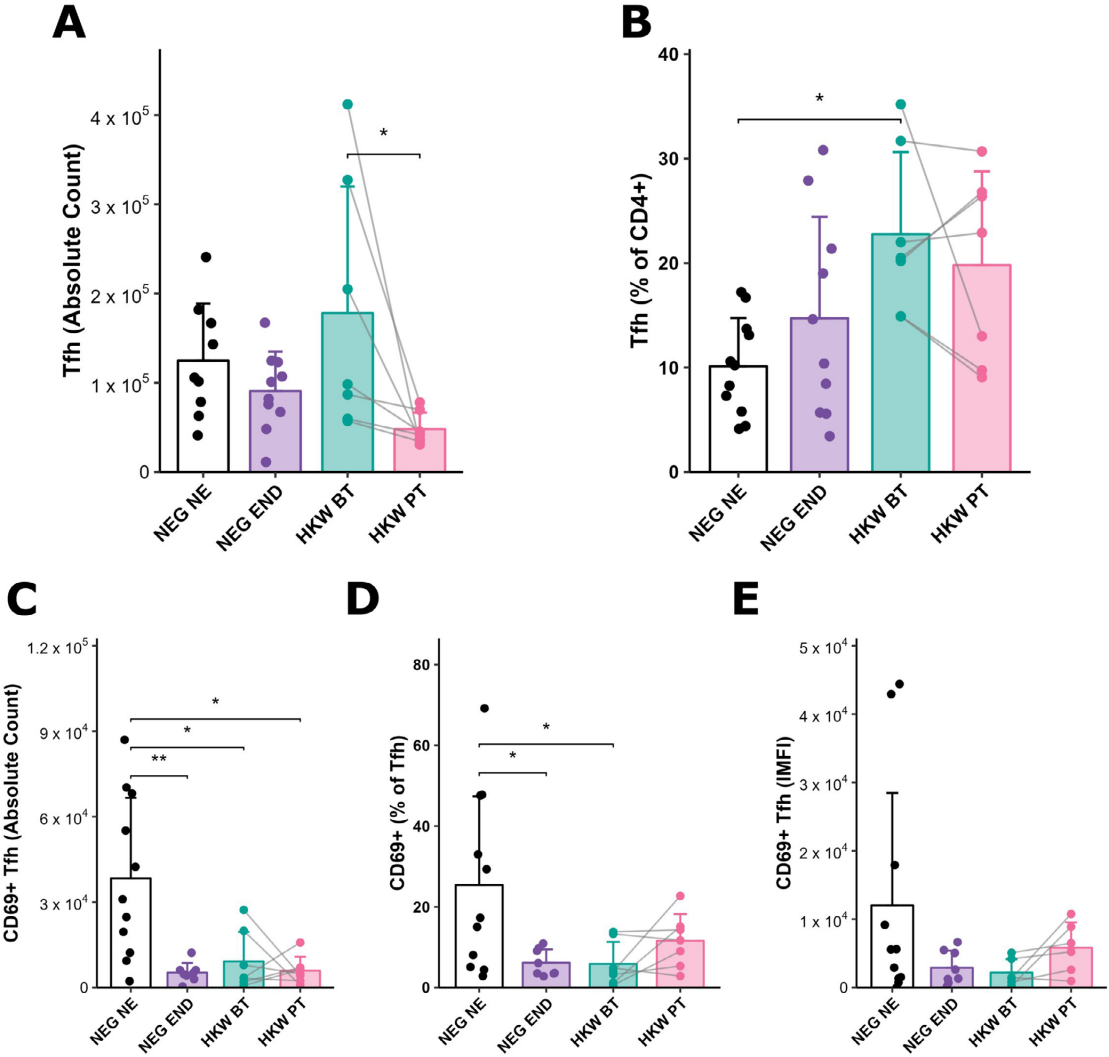
Hookworm infection expands the Tfh cell population, which displays a suppressed activation profile in individuals from endemic areas. (A) Absolute numbers and (B) percentages of CD4^+^CXCR5^+^STAT3^+^ Tfh cells. (C–E) Activation of Tfh cells assessed by CD69 expression: (C) absolute counts of CD69^+^ Tfh cells, (D) frequency (%) of CD69^+^ Tfh cells, and (E) integrated mean fluorescence intensity (IMFI) of CD69 on Tfh. Data are shown for egg‐negative controls from a non‐endemic area (NEG NE, *n* = 10) and an endemic area (NEG END, *n* = 10), and for individuals infected with hookworms before treatment (HKW BT, *n* = 7) and 30 days after anthelmintic therapy (HKW PT, *n* = 7). For HKW BT and HKW PT groups, connected lines indicate paired samples obtained from the same individuals before and after treatment. *p < 0.05; **p < 0.01; ***p < 0.001.

To evaluate whether Tfh cells expand in response to hookworm infection, CD4^+^CXCR5^+^STAT3^+^ cells were quantified in individuals from endemic and non‐endemic areas. Infected individuals before treatment (HKW BT) displayed higher Tfh cell numbers compared with those assessed after treatment (HKW PT) (Figure 4A). Furthermore, the frequency of Tfh cells was significantly elevated in the HKW‐BT group when compared with NEG NE (Figure 4B). These findings indicate that hookworm infection promotes Tfh cell expansion, while individuals from non‐endemic areas harbor lower frequencies of these cells. Notably, Tfh cell numbers declined after anthelmintic treatment, highlighting the infection‐dependent modulation of this population.

We next assessed the activation state of Tfh cells by measuring the expression of the early activation marker CD69. CD69 is rapidly induced upon antigen recognition and has been implicated in sustaining Tfh–B cell interactions within germinal centres [10]. In contrast to the expansion pattern, NEG NE individuals exhibited the highest absolute numbers and frequencies of CD69^+^ Tfh cells when compared to endemic controls (NEG END), infected individuals before treatment (HKW BT), and post‐treatment individuals (HKW PT) (Figure 4C). Similarly, the frequency of CD69+ Tfh cells was significantly higher in the NEG NE group compared to both NEG END and HKW‐BT groups (Figure 4D). Integrated Mean fluorescence intensity (IMFI) analysis further supported this pattern, with NEG NE individuals showing the strongest CD69 signal (Figure 4E). Collectively, these findings suggest that although hookworm infection drives the expansion of Tfh cells, chronic exposure in endemic regions is associated with suppressed Tfh activation. Notably, treatment tended to partially restore Tfh activation in infected individuals, suggesting that parasite clearance may reset activation profiles towards those observed in non‐endemic controls.

### 3.3 A Coordinated IL‐9, IL‐10, and Th17 Response Defines the Post‐Anthelmintic Treatment Immune Signature

**FIGURE 5:**
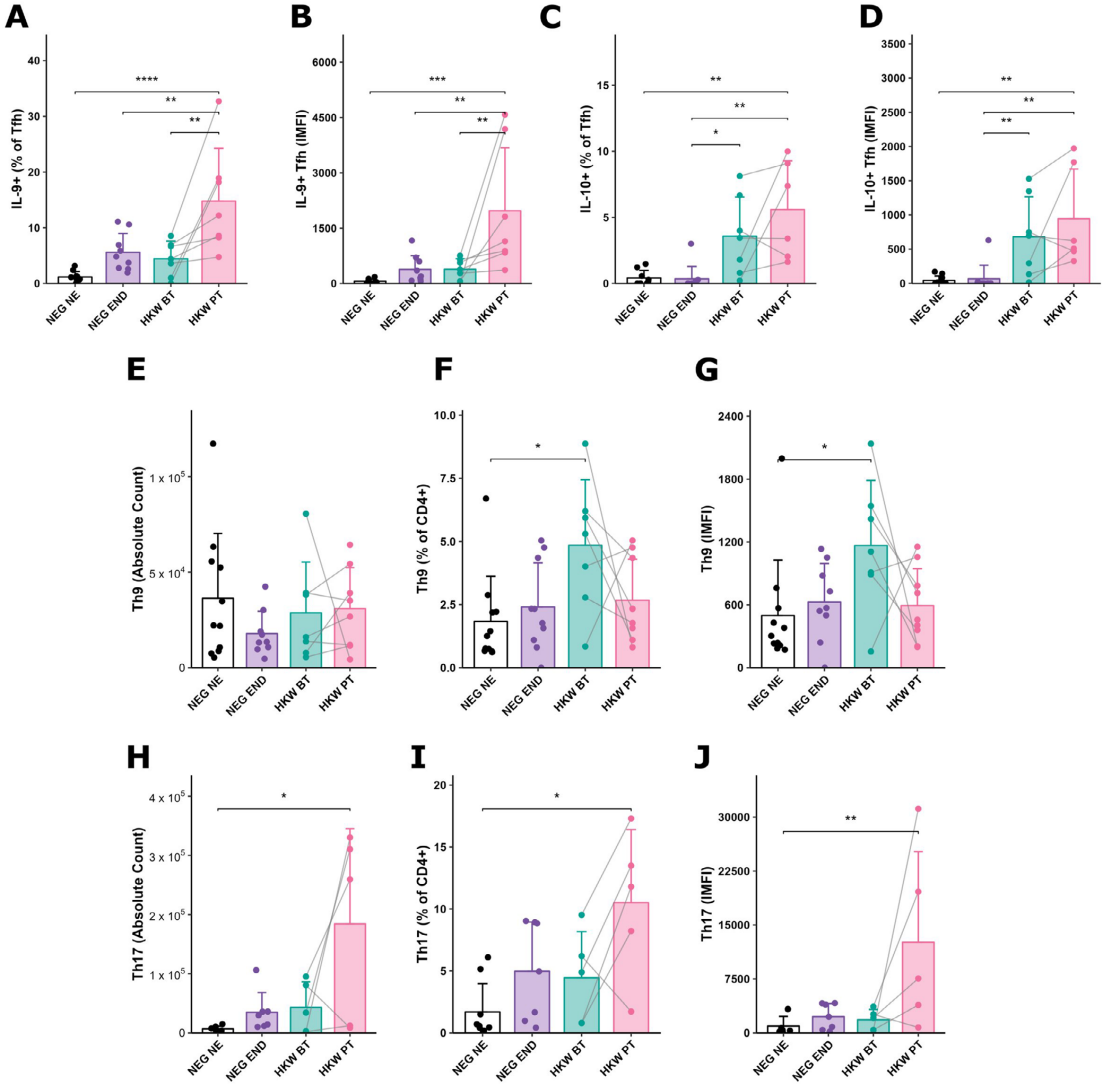
A coordinated IL‐9, IL‐10, and Th17 response defines the post‐treatment immune signature. Tfh cytokine profile and lymphocyte subsets across study groups. (A–D) Tfh cell cytokine expression: (A) percentage of IL‐9^+^ Tfh cells, (B) IMFI of IL‐9 in Tfh, (C) percentage of IL‐10^+^ Tfh cells, and (D) IMFI of IL‐10 in Tfh. (E–G) CD4^+^IL‐4^−^IL‐9^+^IL‐10^+^ population: (E) absolute counts, (F) percentage, and (G) mean fluorescence intensity (MFI) of IL‐9 in this subset. (H–J) Th17 compartment: (H) absolute counts, (I) percentage, and (J) MFI of IL‐17 in CD4^+^CCR6^+^STAT3^+^IL‐17^+^ (Th17) cells. Data are shown for egg‐negative controls from a non‐endemic area (NEG NE, *n* = 10) and an endemic area (NEG END, *n* = 10), and for individuals infected with hookworms before treatment (HKW BT, *n* = 7) and 30 days after anthelmintic therapy (HKW PT, *n* = 7). For HKW BT and HKW PT groups, connected lines indicate paired samples obtained from the same individuals before and after treatment. *p < 0.05; **p < 0.01; ***p < 0.001.

To investigate the functional profile of T helper cells, we first assessed intracellular cytokine expression in the Tfh population. Tfh cells can display functional heterogeneity, and while IL‐4 was virtually undetectable (data not shown), we observed dynamic regulation of IL‐9 and IL‐10. The frequency of IL‐9^+^ Tfh cells was higher in treated individuals (HKW PT) compared to all other groups, especially relative to NEG NE controls (Figure 5A). This was further corroborated by the higher IMFI in HKW PT, which was increased from all other groups (Figure 5B). These data suggest that IL‐9 may play an active role in sustaining Tfh responses after parasite clearance. In contrast, IL‐10 expression followed a different pattern. The percentage of IL‐10^+^ Tfh cells was significantly higher in hookworm‐infected individuals, both before and after treatment, when compared to non‐infected individuals (Figure 5C). Moreover, MFI levels of IL‐10 were already elevated prior to treatment and remained high post‐treatment (Figure 5D), indicating a sustained regulatory phenotype. This pattern highlights a dual role for Tfh cells, where sustained IL‐10 production provides a regulatory environment while treatment specifically boosts IL‐9 expression.

Beyond the Tfh compartment, we investigated other IL‐9–producing subsets. We identified a population of CD4^+^IL‐4^−^IL‐10^+^IL‐9^+^ cells that, unlike the Tfh‐derived IL‐9 response, was more prominent during active infection. Despite no changes in absolute number (Figure 5E), a higher percentage of these cells (Figure 5F), with increased IL‐9 expression (Figure 5G), was detected in infected individuals before treatment (HKW BT) compared with non‐endemic controls (NEG NE). This suggests that active hookworm infection promotes the polarisation of non‐Tfh cells into an IL‐9 and IL‐10 co‐producing phenotype.

In addition to the modulation of the IL‐9 and IL‐10 axis, we explored whether the Th17 response was also affected. We quantified CD4^+^CCR6^+^STAT3^+^IL‐17^+^ cells and found that, similar to the Tfh‐derived IL‐9 response, the Th17 signature was strongest after parasite clearance. Post‐treatment individuals (HKW PT) showed significantly higher absolute numbers, frequencies, and IL‐17 expression (IMFI) of Th17 cells compared with non‐endemic controls (NEG NE) (Figure 5H–J). Although differences across the remaining groups did not reach statistical significance, HKW PT tended to display the highest Th17 counts and IL‐17 MFI among all conditions.

Collectively, these data reveal that anthelmintic treatment drives a distinct and coordinated immune signature. While infection promotes IL‐10‐dominated Tfh and IL‐9^+^IL‐10^+^ CD4^+^ subsets with regulatory potential, the post‐treatment landscape is defined by three key features: a dramatic increase in IL‐9 production by Tfh cells, sustained high levels of regulatory IL‐10 within the same Tfh population, and an amplification of the Th17 response. This suggests that parasite clearance triggers a complex immune program aimed at supporting barrier immunity and parasite control.

### 3.4 Multivariate Analysis Reveals Distinct Immune Signatures Shaped by Endemic Exposure and Treatment

**FIGURE 6:**
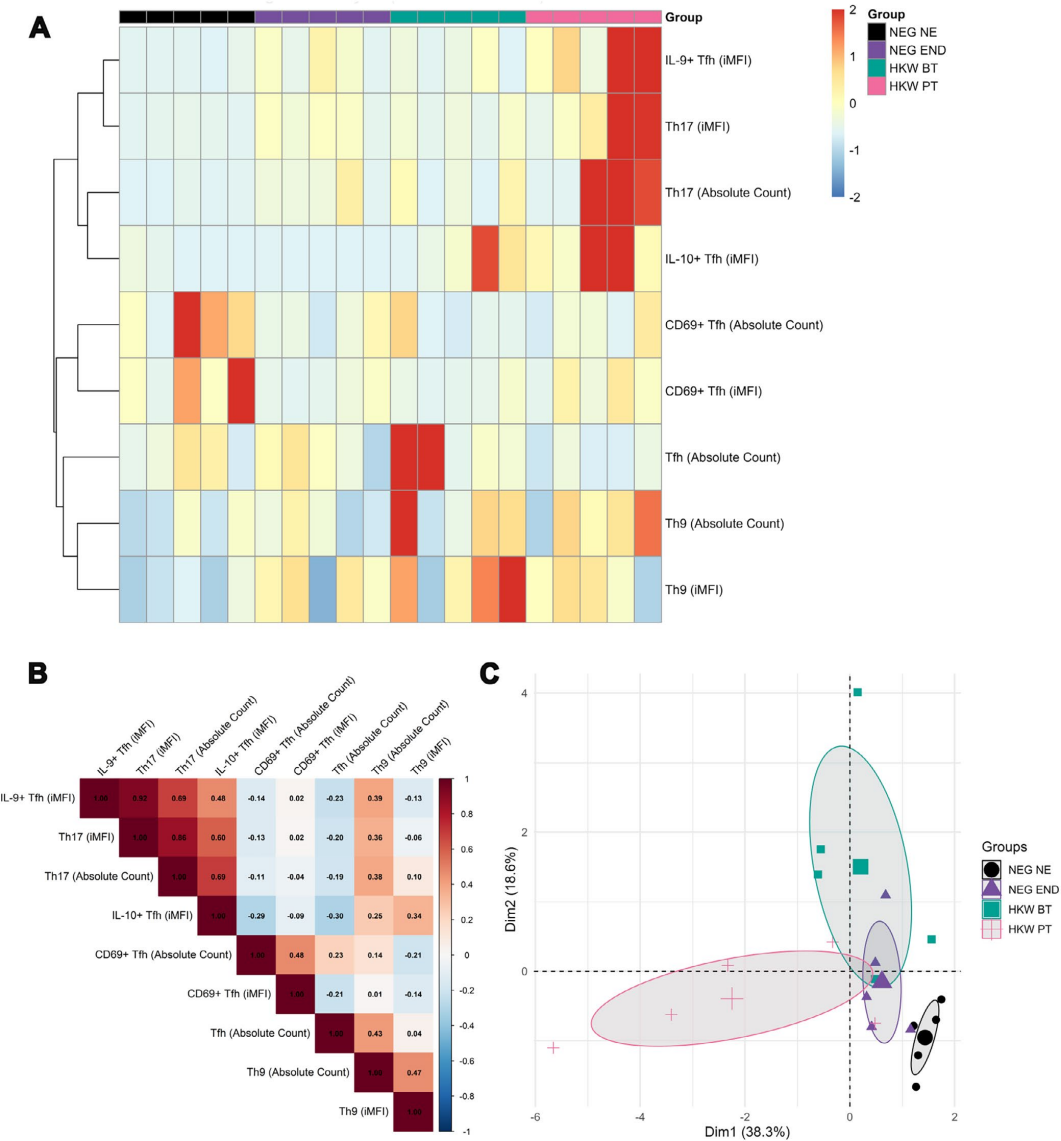
Integrated multivariate analysis reveals distinct immune signatures driven by endemic exposure and treatment. (A) Heatmap with hierarchical clustering of key immune parameters across individuals, showing distinct profiles for each study cohort. (B) Correlation matrix (correlogram) of Pearson correlations between immune parameters. Note the strong positive correlation between Tfh‐derived IL‐9, IL‐10, and Th17 markers, which characterise the post‐treatment group. (C) Principal Component Analysis (PCA) plot of the complete dataset, showing the immunological separation between cohorts. Ellipses represent 95% confidence intervals.

To obtain an integrated view of the immune response, we applied a series of multivariate analyses. First, a heatmap with hierarchical clustering revealed distinct immunological patterns across the four groups (Figure 6A). Individuals from the non‐endemic area (NEG NE) were characterised by higher expression of the early activation marker CD69 on Tfh cells. In contrast, uninfected (NEG END) and infected individuals (HKW BT) from the endemic area displayed overlapping profiles, enriched for Tfh and IL‐9^+^ features. Notably, the post‐treatment group (HKW PT) exhibited a unique signature, defined by the coordinated increase in Tfh‐derived IL‐9, Tfh‐derived IL‐10, and the Th17 response.

To further explore the relationships between these key markers, we generated a correlation matrix. This revealed a strong, significant positive correlation between Tfh‐derived IL‐9, Tfh‐derived IL‐10, and parameters of the Th17 cell population, confirming that these factors form a coordinated network that characterises the post‐treatment phase (Figure 6B).

Finally, to visualise the overall immunological separation between the cohorts based on all measured parameters, we performed a Principal Component Analysis (PCA)b(Figure 6C). NEG NE individuals formed a distinct cluster, highlighting their unique baseline immune profile. Confirming the heatmap analysis, the NEG END and HKW BT groups showed overlapping distributions, suggesting that constant antigen exposure in endemic settings fosters an immune environment that resembles active infection. Remarkably, the HKW PT group formed its own separate cluster, distinct from all others, underscoring how profoundly treatment reshapes the immune landscape.

Together, these data indicate that living in an endemic area shapes immune responsiveness in a way that persists independently of active infection, while anthelmintic treatment drives polarisation toward a distinct immune state characterised by a coordinated signature of IL‐9, IL‐10, and Th17 responses.

## 4 Discussion

Several decades ago, efforts to investigate the pattern of immune response in individuals infected with soil‐transmitted helminths, particularly hookworms, led to numerous studies highlighting the immunomodulatory and regulatory roles of these parasites [23, 24, 25, 26, 27] including considerations of the infection stage [28, 29] and interactions between different helminths [30]. However, the influence of the environment in which individuals reside on this response has received relatively little attention.

In this study, we examined circulating T cell phenotypes and cytokine signatures in adults differing by residence (endemic versus non‐endemic areas) and by hookworm infection status (uninfected, infected before treatment, and 30 days after anthelmintic therapy). Our data reveal interrelated observations. First, living in an endemic area is associated with a baseline immune state that resembles the state of actively infected individuals; second, infection with hookworms is accompanied by measurable changes in circulating Tfh‐like cells and other lymphocyte populations; and third, anthelmintic treatment provokes a pronounced reshaping of the immune profile that is distinguished by increased IL‐9 and IL‐10 within Tfh compartments together with a relative elevation of Th17 markers. Taken together, these findings indicate that exposure to helminth antigens triggers a regulated immunological landscape, and that parasite clearance induces a coordinated program of helper and regulatory responses.

Notably, haematological results showed no significant increase in eosinophils, which is inconsistent with literature findings that emphasise the importance of eosinophilic participation in defence against helminths [31, 32]. As previously shown [33, 34, 35], the populations of monocytes and lymphocytes were proportionally higher in individuals from endemic areas, whether infected or not, with significantly greater numbers only in the infected group compared to individuals from non‐endemic areas.

Regarding Tfh cells, to the best of our knowledge, this is the first investigation into their role in defence against hookworms. Most studies examining Tfh cells in this context focus on *Schistosoma* sp. infections [13], and due to this scarcity of data, it is crucial to explore the versatility of Tfh cells in infections with other helminths. Circulating Tfh cells are known to adopt different phenotypes, designated “Tfh‐Thx‐like cells” due to the expression of interleukins and transcription factors that resemble Th1 (cTfh1), Th2 (cTfh2), and Th17 (cTfh17) phenotypes [13]. Rajamanickam et al. (2018) [36] demonstrated that during *Strongyloides stercoralis* infection, levels of IL‐9 and IL‐4 were significantly increased compared to uninfected individuals. These cytokines together define a Th2 profile (or cTfh2). In contrast, this study did not detect IL‐4 expression in Tfh cells but did identify IL‐9 and IL‐10, allowing speculation about the presence of a “cTfh9” subpopulation. IL‐9 has been implicated in promoting aspects of humoral immunity and mucosal defence in other models, and its upregulation could therefore reflect a beneficial remodelling of Tfh help towards improved antibody quality [37]. IL‐10 within Tfh cells could serve to restrain excessive inflammation and to skew antibody class switching in a manner that minimises tissue damage while maintaining humoral defence [38]; the coexistence of IL‐9 and IL‐10 is thus consistent with a program that couples enhanced helper capacity with regulatory control.

Circulating cTfh cells represent a small fraction of peripheral CD4^+^ T cells in healthy individuals. Studies defining cTfh cells as CXCR5^+^CD4^+^ lymphocytes, or using additional activation markers such as PD‐1 or ICOS, generally report frequencies in the low single‐digit range, although substantial variation exists depending on the phenotypic definition employed and the population studied [39, 40]. This limitation is even more pronounced for rare functional subsets, such as IL‐9–producing Tfh or Th9‐like CD4^+^ populations, for which standardised baseline ranges have not been defined. In this context, the use of non‐endemic negative individuals from the same country, matched for age and sex, provides the most appropriate biological comparator for defining homeostatic immune profiles and for interpreting infection‐ and treatment‐associated deviations.

An important observation in the post‐treatment group (HKW PT) was the robust reactivation of the immune response. We propose that this heightened activity arises from converging mechanisms. The anthelmintic‐induced death of adult worms releases a surge of somatic antigens, which, combined with the cessation of parasite‐mediated immunosuppression, such as the downregulation of IL‐10 by excretory/secretory products, effectively shapes effector cells [34, 41, 42]. Furthermore, the intrinsic immunomodulatory properties of albendazole warrant consideration. Beyond their direct effects on cellular responses and cytokine kinetics [43, 44], benzimidazoles may influence the immune landscape indirectly through the gut microbiome. Recent evidence demonstrates that successful albendazole treatment induces significant shifts in gut microbiota composition and diversity [45]. Given the pivotal role of the microbiota in shaping T helper cell plasticity, particularly Th17 differentiation, these treatment‐associated microbial perturbations may synergize with the antigen release from dying worms to drive the distinct Th17 and IL‐9 profiles observed 30 days post‐treatment.

We also identified a population of non‐Tfh CD4 + IL‐4‐IL‐9 + IL‐10+ cells that expanded during active infection. In contrast to classical Th9 cells, which typically decline post‐treatment [36], the persistence of this specific subset in endemic residents suggests a long‐lived memory phenotype generated by chronic exposure. This aligns with the observed suppression of the early activation marker CD69 in Tfh cells from endemic individuals [35]. This dampened basal activation likely reflects a functional adaptation to the high antigenic load inherent to endemic regions, preventing pathological inflammation through a mechanism of immune hyporesponsiveness [46].

Regarding the Th17 axis, the post‐treatment upregulation of IL‐17A appears to reflect a restoration of mucosal barrier integrity. While murine models have linked mixed Th2/Th17 responses to protection against reinfection [47], our data indicate that in humans, this signature is most prominent following parasite clearance. The simultaneous elevation of IL‐9, IL‐10, and IL‐17 suggests a coordinated restorative process: IL‐9 and IL‐17 promote tissue repair and resistance, while sustained IL‐10 levels mitigate potential immunopathology [48].

Finally, it is important to acknowledge that the rigorous selection criteria applied in this study resulted in a reduced final sample size relative to the initial screening of 1500 individuals. However, this strict selection process, aimed at excluding confounding variables and ensuring sample viability, was essential to ensure that the observed immunological profiles were specifically attributable to hookworm infection and endemic exposure, rather than other environmental variables. Furthermore, while rigorous clinical screening was employed to exclude individuals with severe nutritional deficiencies or comorbidities, Body Mass Index (BMI) was not systematically quantified for all participants. Although we do not anticipate that minor variations in BMI significantly influenced the specific T‐cell phenotypes observed, we acknowledge this as a limitation. Future studies investigating metabolic‐immune interactions in helminth infections should include precise BMI measurements to further refine these associations.

In conclusion, this study highlights the distinct, tolerogenic immune profile shaped by continuous antigen exposure in endemic environments. Our findings demonstrate that hookworm treatment does not simply reset the immune system to a naïve state but rather initiates a complex program of barrier‐protective and regulatory responses. These insights reinforce the critical importance of including endemic controls in immunological studies, as pre‐existing regulatory networks may fundamentally alter biomarker interpretation and vaccine efficacy.

## Author Contributions

**Yvanna Louise Di Christine Oliveira:** conceptualization; data curation; formal analysis; investigation; methodology; writing – original draft; writing – review and editing. **Marcelo Eduardo Cardozo:** data curation; investigation; writing – review and editing. **Luisa Mourão Dias Magalhães:** conceptualization; investigation; methodology; writing – review and editing. **Carlos Thailan de Jesus Santos:** data curation; investigation. **Ramayana Morais de Medeiros Brito:** data curation; investigation; methodology. **Luciana Maria de Oliveira:** data curation; investigation; methodology. **Ana Carolina Amado Gomes:** investigation. **Vinícius Torres Castro Campos:** data curation; investigation; methodology. **Ricardo Toshio Fujiwara:** conceptualization; formal analysis; funding acquisition; project administration. **Silvio Santana Dolabella:** conceptualization; funding acquisition; investigation; methodology; project administration; supervision; writing – review and editing. **Lilian Lacerda Bueno:** conceptualization; funding acquisition; investigation; methodology; project administration; supervision; writing – review and editing.

## Funding

This research was funded by Foundation of Support for Research in the State of Minas Gerais (FAPEMIG/Brazil, grant # APQ‐4035/17), Rede Mineira de Imunobiológicos (RED‐00067‐23), and National Council for Scientific and Technological Development (CNPq/Brazil, grants # 402693/2021‐3 and # 403278/2023‐6).

## Disclosure

The authors have nothing to report.

## Conflicts of Interest

The authors declare no conflicts of interest.

## Supporting information


**Table S1:** Demographic, socioeconomic, and clinical characteristics of the study population.

## Data Availability

The data that support the findings of this study are available from the corresponding author upon reasonable request.
