# Supplementary material for: Comparison Between Phenotypic Profile and Functional Aspects of IL‐9‐Producing Lymphocytes, Th17 and Tfh of Individuals From Endemic and Non‐Endemic Areas for Hookworm Infection
Source: Parasite Immunol. 2026 Mar 13;48(3):e70070. doi: 10.1111/pim.70070 (PMC12983440; doi:10.1111/pim.70070)
Supplement: Supplementary file 1 — Table S1: Demographic, socioeconomic, and clinical characteristics of the study population. [file PIM-48-e70070-s001.docx]

**Table S1. Demographic, socioeconomic, and clinical characteristics of the study population.**

| **Characteristics** | **NEG NE** | **NEG END** | **HKW** | **Total** |
| --- | --- | --- | --- | --- |
| **Total Participants (N)** | **13** | **24** | **43** | **80** |
| Mean age | 28.3 | 25.8 | 25.1 | 35.0 ^a^ |
| **Age Groups, n (%)** | | | | |
| 0–18 years (Children/Adolescents) | - | - | - | 29 (36.2%) |
| 19–59 years (Adults) | - | - | - | 49 (61.2%) |
| 60–74 years (Elderly) | - | - | - | 02 (2.5%) |
| **Sex, n (%)** | | | | |
| Male | 6 (46.2%) | 14 (60.0%) | 28 (65.1%) | 34 (42.5%) |
| Female | 7 (53.8%) | 10 (40.0%) | 15 (34.9%) | 46 (57.5%) |
| **Socioeconomic & Environmental** **Factors^b^** | **(n=0)** | **(n=7)** | **(n=28)** | **(n=35)** |
| Rural residence, n (%) | N/A | 6 (85.7%) | 27 (96.4%) | 33 (94.3%) |
| Unpaved streets, n (%) | N/A | 6 (85.7%) | 27 (96.4%) | 33 (94.3%) |
| Indoor bathroom, n (%) | N/A | 6 (85.7%) | 24 (87.5%) | 30 (85.7%) |
| Untreated tap water, n (%) | N/A | 6 (85.7%) | 23 (82.1%) | 29 (82.8%) |
| **Hygiene & Health History ^b^** | | | | |
| Raw food hygiene (water only), n (%) | N/A | 6 (85.7%) | 23 (82.1%) | 29 (82.8%) |
| Hand washing ^c^, n (%) | N/A | 4 (57.1%) | - | - |
| History of parasitic infection, n (%) | N/A | 2 (28.6%) | 18 (64.3%) | 20 (57.1%) |
| Preventive deworming, n (%) | N/A | 6 (85.7%) | 8 (28.6%) | 14 (40.0%) |
| **Education Level** | | | | |
| Incomplete Primary Education, n (%) | N/A | 5 (71.4%) | 12 (42.9%) | 17 (48.6%) |

**Data are presented as number (percentage) or Mean**. NEG NE: Non-endemic negative control; NEG END: Endemic negative control; HKW: Hookworm infected group.

^a^Overall mean age calculated for the entire cohort.

^b^ Socioeconomic and hygiene data were obtained from a questionnaire applied to a subset of participants (n=35; 7 NEG END and 28 HKW). Participants from the non-endemic area (NEG NE) were not included in this assessment due to distinct urban living conditions.

^c^ Defined as hand washing before meals, after using the toilet, or after handling animals.
